# Targeting reasoning biases in delusions: A pilot study of the Maudsley Review Training Programme for individuals with persistent, high conviction delusions

**DOI:** 10.1016/j.jbtep.2011.03.001

**Published:** 2011-09

**Authors:** Helen Waller, Daniel Freeman, Suzanne Jolley, Graham Dunn, Philippa Garety

**Affiliations:** a Institute of Psychiatry, King’s College London, P.O. Box P077, 16 De Crespigny Park, London SE5 8AF, UK; b Oxford University, Department of Psychiatry, Warneford Hospital, Oxford OX3 7JX, UK; c University of Manchester, Health Methodology Research Group, Jean McFarlane Building (First Floor), Oxford Road, Manchester M13 9PL, UK

**Keywords:** Psychosis, Delusions, Cognitive Behaviour Therapy, Reasoning, Jumping to Conclusions

## Abstract

Delusions are often resistant to change, persisting despite successful antipsychotic treatment or Cognitive Behavioural Therapy. This study aimed to target reasoning processes, particularly the ‘Jumping to Conclusions’ (JTC) bias and belief flexibility, which are thought to play a part in maintaining delusional conviction.

13 participants with a diagnosis of psychosis and high levels of conviction in their delusions completed a one-off computerised training package, lasting approximately 1.5 h. Outcomes were assessed at baseline, pre-intervention (two weeks later), post-intervention (immediately after completing the training) and at 1 month follow-up.

The package was well received by participants. There were improvements in JTC, belief flexibility and delusional conviction between pre- and post-intervention measures. Controlled studies powered to detect changes in key outcomes are warranted in order to evaluate the efficacy of the programme.

## Highlights

► Delusions are resistant to change. ► Reasoning biases are thought to contribute to the maintenance of high delusional conviction. ► We have devised a computerised training programme targeting the ‘Jumping to Conclusions’ (JTC) bias and belief flexibility. ► In a case series evaluation, participants completing the training showed improvements in JTC, belief flexibility and delusional conviction. ► Larger-scale, controlled studies of this approach are warranted.

## 1 Introduction

Delusions are particularly resistant to change, often persisting despite antipsychotic treatment (Craig et al., 2004; Mizrahi et al., 2006). Recent meta-analytical studies of Cognitive Behavioural Therapy for psychosis (CBTp) have found small- to medium-sized beneficial effects on residual positive symptoms (Wykes, Steel, Everitt, & Tarrier, 2008). It has been argued that the development of more targeted interventions, based upon theoretical models of single symptoms, may be a way forward (Garety et al., 2008). Examples of interventions using this approach have found promising results for command hallucinations (Trower et al., 2004) and persecutory delusions (Foster, Startup, Potts, & Freeman, 2010). It is hypothesised that an intervention targeting reasoning biases, including the ‘Jumping to Conclusions’ (JTC) bias and belief flexibility will reduce patients’ conviction in their delusional beliefs.

Experimental research has consistently found that people with delusions are more likely to jump to conclusions than non-delusional controls (see reviews by Fine, Gardner, Craigie, & Gold, 2007; Freeman, 2007; Garety & Freeman, 1999). That is, in uncertain situations they gather less information before reaching a conclusion: they show a specific ‘data gathering bias’. The tendency to JTC appears to be specific to the presence of delusions rather than other psychotic symptoms or diagnosis (Freeman, Pugh, & Garety, 2008; Garety et al., 2005; Peters, Thornton, Siksou, Linney, & McCabe, 2007), and it has been associated with the presence of inflexible beliefs (Garety et al., 2005) and higher delusional conviction (Broome et al., 2007; Freeman et al., 2008; Garety et al., 2005). Garety et al. (2005) report evidence for an association between JTC and delusional conviction, which they found to be mediated by the related reasoning process of belief (in)flexibility. Belief flexibility has been defined as, ‘*the metacognitive capacity of reflecting on one’s own beliefs, changing them in the light of reflection and evidence, and generating and considering alternatives’* (Garety et al., 2005, p.374).

A recent study by Lincoln, Ziegler, Mehl, and Rief (2010) reported evidence that the JTC bias may be reduced in delusional patients, to some extent, following provision of feedback, highlighting errors and successes in their decision-making. Training patients in the use of ‘good’ reasoning strategies may therefore be one way of reducing the JTC bias and impacting on delusional ideation. To date only a small number of studies have aimed to manipulate reasoning biases. Moritz and Woodward (2007a, b) report the development of a group-based ‘Metacognitive Training’ (MCT) package targeting a number of cognitive processes which included JTC. Training is delivered over eight sessions, each lasting approximately 45–60 min. In two small pilot studies (Aghotor, Pfueller, Moritz, Weisbrod, & Roesch-Ely, 2010; Moritz & Woodward, 2007a) the intervention was found to be well received by participants and there were indications of a decline in both JTC and positive symptoms, although more rigorous evaluations are warranted. Ross, Freeman, Dunn, and Garety (2009) were the first group to report the results of an intervention specifically targeting the JTC data gathering bias. They were interested in the short-term effects of a brief individually-delivered intervention. Thirty-four high conviction delusional patients took part in the study, randomly assigned to either the experimental group, who completed the computerised reasoning training lasting approximately 45 min, or the control group, who completed cognitive tasks unrelated to reasoning. The training package aimed to ‘target data gathering, generation and consideration of alternative ideas and the use of confirmatory and disconfirmatory evidence’ (Ross et al., 2009, p. 4). It comprised three modules: two significantly adapted from Moritz and Woodward’s (2007a) group MCT package, and one newly developed. All three modules involved neutral, abstract tasks, with no reference to delusions or potentially delusion-related materials. Following training, participants in the experimental group showed a significant improvement in data gathering (JTC) in comparison with the control group, with an effect size of 0.4 (Cohen’s d). However, this was most prominent in those who did not JTC on baseline measurements, suggesting that the bias is relatively robust and that lengthier training may be needed to improve reasoning in those who do JTC. In the initial study the focus was simply on changing the reasoning bias, however it is of interest that a small number of participants in the experimental group also reported improvements in belief flexibility (*n* = 4) and delusional conviction (*n* = 3) following training, whereas only one of the control participants showed similar improvements and only in belief flexibility. Overall, these findings suggest that reasoning training may hold promise in improving key outcomes in delusions. However, clearly more work is needed in order to further develop the training programme, with the aim of impacting on JTC, belief flexibility and delusional conviction.

The present study aimed to extend the work of Ross et al. (2009) through the development of a significantly modified training programme. Delusion-relevant material was added, emphasising the link between JTC, belief flexibility and delusional experience, with the aim of encouraging generalisation to participants’ own beliefs. Interactive components were included, with the aim of encouraging sustained interest. A handout was provided to summarise key aspects, to act as a memory aid and to facilitate continued learning. Finally, a follow-up session was included in order to evaluate the impact of the training over time and on real-life situations where JTC might previously have occurred and played a role in maintaining delusions. As a result the training was lengthened to 1.5 h.

We report the results of a pilot study examining the acceptability and feasibility of the programme, and its impact on reasoning, belief flexibility and delusional conviction. This type of design is recommended in early-stage development of new clinical interventions before moving to controlled trials (Medical Research Council, 2000, 2008). It was hypothesised that following training participants would show an increase in data gathering (reduced JTC), an increase in belief flexibility and a decrease in conviction in their primary delusion, which would be sustained at two-week follow-up. We aimed to produce a short-term change in reasoning style even in those with the strongest JTC reasoning bias. In contrast, it was hypothesised that there would be no change in these outcomes over an initial two-week baseline period, when no intervention occurred.

## 2 Method

### 2.1 Design

An A-B design was used, incorporating two baseline and two post-intervention measurements, completed over three meetings. Participants completed key outcome measures at all time points. The Positive and Negative Syndrome Scales (PANSS, positive scale only, Kay, Opler, & Lindenmayer, 1989) were administered at baseline in order to establish the primary delusion, to allow completion of further assessments. At baseline participants also completed measures of positive psychotic symptoms (Psychotic Symptoms Rating Scale (PSYRATS), Haddock, McCarron, Tarrier, & Faragher, 1999), mood (Depression Anxiety Stress Scale, Abbreviated Version (DASS-21), Lovibond & Lovibond, 1993) and intellectual functioning (Wechsler Abbreviated Scale of Intelligence (WASI), Wechsler, 1999). All assessments were completed by a Doctoral Clinical Psychologist in Training, with a background in research (HW), who was also the therapist delivering the programme to participants.

### 2.2 Participants

Psychiatrists and care-coordinators working in the South London and Maudsley NHS Foundation Trust were approached and asked to identify individuals meeting the following inclusion criteria: a case note diagnosis of a schizophrenia spectrum disorder; currently experiencing paranoid delusions (defined using the criteria described by Freeman & Garety, 2000); delusions present for at least six months. Those with a difficulty in spoken or written English, organic impairment, a learning difficulty or profound visual impairment were excluded from the study. In order to ensure recruitment of a group with high delusional conviction, those with less than 75% self-rated conviction were also excluded from the study. A total of 23 people were referred to the study: two did not wish to take part, four were unable to be contacted, and three were excluded due to low levels of conviction in their delusions. A total of 14 people were recruited for the study: six from local community mental health teams, and the remaining nine were recruited via a research register compiled by a local specialist team dealing with psychosis. This number was deemed large enough to gain an understanding of the potential feasibility and acceptability of the training programme. Research into the development of new CBT-based interventions frequently report similar sample sizes in pilot studies, before moving to a larger-scale trial (e.g. Dobkin, Allen, & Menza, 2007; Reid, Otis, Barry & Kerns, 2003; Szigethy et al., 2004). Thirteen participants completed the study and one participant did not attend meetings two and three so was not included in the analysis. Participants were paid £30 for their time and any expenses incurred over the study.

### 2.3 Primary outcomes

#### 2.3.1 Data gathering/‘Jumping to Conclusions’

The Probabilistic Reasoning Task (60:40 version), also known as the ‘Beads Task’ (Garety, Hemsley, & Wessely, 1991) was used to assess JTC reasoning style. In this task, participants were shown two jars of beads: a ‘mainly red’ jar with 60 red beads and 40 blue beads and a ‘mainly blue’ jar with 60 blue beads and 40 red beads (in order to reduce possible practice effects, the colours of the beads were changed at each different time point). The jars were subsequently hidden from view. Participants were told that one of the jars would be ‘selected at random’ and beads would be drawn from that jar. In fact the order of the beads shown is pre-determined in order to standardise the procedure. Participants were informed that they could see as many beads as they wish (up to a total of 20) before making a decision about which jar they have come from, and to only make a decision when they were sure. The number of beads requested before making a decision was recorded and used as the key outcome variable. Participants were rated as showing the JTC bias if they made a decision after seeing two or fewer beads, as described by Garety et al. (2005).

#### 2.3.2 Belief flexibility

Three items were used to assess belief flexibility. The first two items were from (or adapted from) the ‘Belief Maintenance’ scale of the Maudsley Assessment of Delusions Schedule (MADS: Buchanan et al., 1993), both of which have good reported inter-rater reliability (Buchanan et al., 1993). Where more than one delusional belief had been identified in the PANSS interview, participants were asked to state which was most strongly held. Only the most strongly held delusion was examined and rated in subsequent sessions.

*Possibility of Being Mistaken (PBM)*: In the first item, which was the key outcome for belief flexibility, participants were asked to rate how likely it is that they are mistaken about their delusional ideas on a visual analogue scale, ranging from 0, indicating no possibility of being mistaken, to 100, indicating a strong possibility that they are mistaken. This method of rating on a 100-point scale was adapted from the original yes/no response in order to provide continuous data.

*Reaction to Hypothetical Contradiction (RTHC)*: For the second item, participants were given a hypothetical scenario involving evidence which was clearly inconsistent with the delusion and were asked how they would react. Participants’ responses were classified as ‘showing flexibility’ (either changing their level of conviction or dismissing the delusion) or ‘showing inflexibility’ (ignoring or rejecting the relevance of the contradictory information).

*Explanations of Experiences (EoE)*: The third item was taken from the Explanation of Experiences Assessment (Freeman et al., 2004), which is a structured interview used to assess whether a person can think of alternative explanations for the evidence cited in support of the delusional belief. The number of alternative explanations was recorded. Freeman et al. (2004) reported excellent stability of the measure in those with similar levels of delusional conviction at three months follow-up, and good concurrent validity.

#### 2.3.3 Delusional conviction

Current conviction in participants’ primary delusion was measured on a visual analogue scale, ranging from 0 to 100%, where 0% indicated no conviction in the belief and 100% indicated total conviction.

### 2.4 Participant feedback

Feedback was sought on participants’ views of the training, including whether they saw any relevance to their own experiences and what they felt they had learnt from the training.

### 2.5 The Maudsley Review Training Programme

The computerised programme was developed on Microsoft PowerPoint and then transferred to a Real BASIC programme to incorporate the interactive elements. It comprised a general introduction to JTC and five training tasks (see below). It was designed to be completed together with a therapist, who emphasised key messages and provided feedback on participants’ comments, for example by reinforcing useful insights and normalising JTC. This was summarised, with participants’ permission, in a handout to be taken away at the end of the session. No explicit mention of psychosis or direct challenging of beliefs was included. However, in order to increase relevance to delusional experience, video clips and scenarios, with the potential for paranoid interpretation, were used.

#### 2.5.1 Introduction

The introduction described the concept of JTC, highlighting that everyone jumps to conclusions sometimes, especially in unclear or confusing situations, and that this can lead us to make mistakes. Participants were encouraged to think about any times when they might have jumped to conclusions.

#### 2.5.2 Task One: ‘What’s the picture?’

Task One was designed to introduce the idea that it can be difficult to understand a situation and reach a conclusion without having all of the information. The main aim was therefore to teach participants to look for more evidence before reaching a conclusion, since it means that they are less likely to make mistakes. The task was adapted from a training module in Moritz and Woodward’s (2007a) MCT package, and was also used in the Ross et al. (2009) study. Participants were shown a series of six pictures, revealed one piece at a time over eight slides. After seeing each piece they were able to either see another piece or decide what the picture was, from a choice of six options. The task was designed so that all options seemed plausible initially, but as more pieces were revealed certain options could be ruled out and it became clearer what the picture actually was.

During the training phase, participants were shown all the pieces they could have seen (i.e. all available evidence) and were given specific advice depending on when they chose to decide. For example, if they decided after seeing only a small number of slides they were told, ‘*That was quite a quick decision! Making decisions without much information can lead to mistakes. Next time try looking at more pieces before coming to a decision*’. Participants were then shown the picture piece by piece and how to eliminate options one at a time.

#### 2.5.3 Task Two: ‘illusions’

Task Two, which was adapted from Ross et al.’s (2009) study, was designed to introduce the idea that things are not always as they first seem, and that sometimes we only see part of the story, which can lead us to JTC and make mistakes. The main aim of the task was to teach participants to slow down in order to think more carefully about other ways of seeing the situation. Participants were shown a series of optical illusions and were asked to state what they saw. During the training phase, participants were encouraged to take their time and think carefully about other possible ways of viewing the picture before coming to a conclusion.

#### 2.5.4 Task Three: ‘first Impressions’

Task Three was designed to highlight that things are not always as they first seem in real life and that not seeing the full story can lead us to JTC and make mistakes, which can impact on our feelings and behaviour. Participants were shown a series of three video clips. The beginning of the clip was a brief extract, designed to make the observer JTC. Participants were asked to state what they thought was happening in the clip and rate how sure they were about this judgement, on a 0–100% scale. During the training phase the full clip was shown, which provided an alternative explanation. Participants were asked to think about whether they jumped to conclusions and whether anyone else in the scene did. Participants were reminded that in real-life things are not always as they first seem and that sometimes we only see part of the story which can lead us to JTC.

#### 2.5.5 Task Four: ‘looking for other possible explanations’

**Fig. 1:**
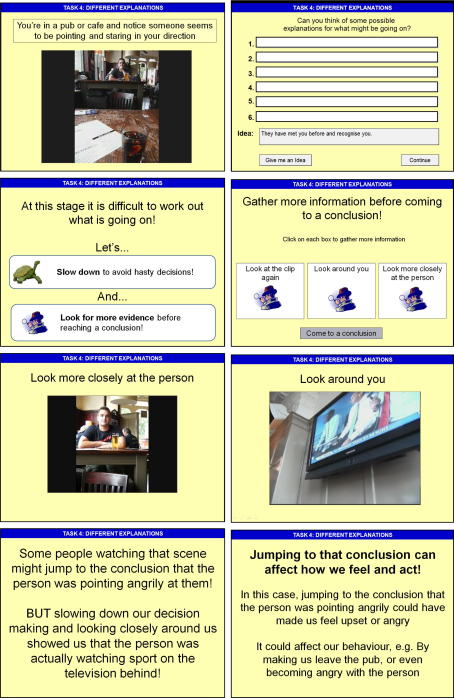
Examples of slides from the Training Programme: Task Four.

Task Four was designed to introduce the idea of thinking flexibly about alternative explanations before reaching a conclusion. Participants were given three scenarios, each with potential for a paranoid interpretation. For example, in scenario one, participants were asked to imagine that they are in a cafe and notice that someone seems to be pointing and staring in their direction. A short video clip illustrating each scenario was presented. After seeing each clip, participants were asked to think about possible explanations for each scenario, and were prompted to think about neutral, positive and negative interpretations (e.g*. ‘What might you think if you were feeling scared or worried?’*). They were also given the option of seeing some ideas, already devised by the researcher (e.g. *‘They have mistaken you for someone else’; ‘They are angry with something you have done’*). During the training phase, participants were given the option to search for more evidence in the form of three further video clips. For example in scenario one they were shown the original clip again, a close-up picture of the person, and finally a clip of the camera panning around to reveal a television showing sports behind them. Participants were then asked to pick which of their explanations seemed most likely. Finally they were told how people might have jumped to conclusions in that situation, given the paranoid interpretation, and suggested that this could impact negatively on their feelings and behaviour. If participants suggested very paranoid interpretations this was gently highlighted and it was suggested that thinking about possible neutral explanations could help them to see the situation differently and feel less threatened as a result. Examples of the slides from Task Four are presented in Fig. 1.

#### 2.5.6 Task Five: ‘JTC summary’

Task Five was designed to be somewhat light-hearted as a final task, but also to refresh the main training points from the previous tasks. Participants were shown four humorous video clips where one character jumps to a conclusion. After seeing the clip, participants were asked to decide which character jumped to conclusions, from a choice of three options. Next they were asked how the character might have avoided jumping to conclusions, and were prompted using the main training points.

### 2.6 Statistical analysis

All statistical analyses were completed using SPSS for Windows, version 15. The main analysis of the three primary outcomes (data gathering (JTC), belief flexibility (PBM only), delusional conviction) was conducted in two stages. In the first stage, a series of one-way within-subjects ANOVAs were calculated for each primary outcome, with planned repeated contrasts. The purpose of this stage was to confirm that there was no change between baseline 1 and 2 measurements and between post-intervention 1 and 2 measurements, as was hypothesised. Following this, mean baseline and mean post-intervention period scores were computed for each of the key outcomes, and a second series of one-way within-subjects ANOVAs was calculated, including effect sizes (Cohen’s d; Dunlap, Cortina, Vaslow, & Burke, 1996), in order to test whether primary outcomes showed change following reasoning training. As a check for sensitivity of the results to skew, non-parametric methods were also used, but the results remained unaltered. For the two non-continuous belief flexibility items (RTHC and EoE), raw change in flexibility was examined.

## 3 Results

### 3.1 Baseline demographic and clinical characteristics

A summary of the demographic and clinical characteristics of participants is provided in Table 1. Participants were from a variety of ethnic backgrounds, but were mainly White and Black African. There was an approximately equal number of males and females, and the mean age was 44.6 years. 46.2% of participants were classified as showing the JTC bias and all reported taking antipsychotic medication. The intellectual functioning of the sample as a whole fell within the low average range (mean full scale IQ = 88.93, sd = 16.43), which is comparable to that reported in research in a similar population (Ross et al., 2009; Sheitman et al., 2000). In terms of clinical characteristics, participants reported high levels of positive symptoms and symptoms of depression, anxiety and stress, all of which were comparable with previous research in a similar population (Haddock et al., 1999; Ross et al., 2009).

### 3.2 Main analysis: stage 1

A summary of mean data on the key outcomes across the four measured time points is displayed in Table 2. Baseline data on all outcomes was comparable with that of Ross et al. (2009). The two, non-continuous, belief flexibility items (RTHC and EoE) and classification of JTC status were not analysed statistically.

A series of planned comparisons between the four time points was conducted for all key outcomes in order to assess differences between specific time points. As predicted, there were no significant differences between scores at baseline 1 and 2 or between post-intervention 1 and 2 on any of the three key outcomes. However, when comparing baseline 2 and post-intervention 1 measurements (i.e. immediately before and after training) there were indications of improvements at post-intervention 1 on the number of beads requested (*F*(1,12) = 4.55, *p* = 0.05), belief flexibility: PBM (*F*(1,12) = 7.36, *p* = 0.02) and delusional conviction (*F*(1,12) = 3.57, *p* = 0.08).

### 3.3 Main analysis: stage 2

Having confirmed that there were no differences within baseline and post-intervention assessment points, average baseline and post-intervention scores were constructed. The results of within-subjects ANOVAs comparing these periods are presented in Table 3. As shown, there was a significant improvement in belief flexibility: PBM (*p* = 0.01) and delusional conviction (*p* = 0.01), and also a trend towards improved reasoning (*p* = 0.07) at post-intervention. Change in data gathering between baseline and post-intervention was also calculated according to participants’ JTC status at baseline. Participants who were classified as showing the JTC bias at baseline requested an additional mean of 1.17 (1.91) beads. Those who did not JTC at baseline requested an additional mean of 0.57 (1.21) beads.

### 3.4 Participant feedback

Mean ratings on the 0–10 visual analogue scales for enjoyment (mean = 8.77, sd = 1.54, range = 5–10), usefulness (mean = 9.38, sd = 1.04, range = 7–10) and interest of the programme (mean = 9.08, sd = 1.44, range = 5–10) were all high. Participants’ responses to open-ended questions about the programme were all positive, and follow-up feedback indicated that they had understood the key training points and a number of participants were able to make links with their own reasoning style. However, there were some participants who did not report changes in their own reasoning style relating to their delusional belief. Participants’ comments were recorded verbatim and examples are provided in Table 4.

## 4 Discussion

The study was designed as an initial test of the efficacy of an enhanced computerised training package targeting reasoning processes in delusions. Thirteen participants with severe and long-standing delusions completed the intervention, all of whom had very high levels of delusional conviction, low levels of belief flexibility and half of whom had a tendency to jump to conclusions. Approximately half of the participants had not responded to CBT in the past, in terms of their delusional experience. The current sample is therefore one that is resistant to change using either traditional CBT for psychosis and/or antipsychotic medication.

The training programme was well received by participants. Ratings of enjoyment, usefulness and interest of the programme were high, and comments made at post-training were very positive. The results of the statistical analyses support the hypothesised impact of training on outcomes. When comparing the average baseline and post-intervention periods there were significant improvements in belief flexibility and delusional conviction, both of which had large effect sizes; and a small effect on reasoning (Cohen, 1992), although this did not reach significance.

As intended, these changes are larger than those reported by Ross et al. (2009) for both delusional conviction and belief flexibility. Furthermore, Ross et al. (2009) achieved a reduction in delusional conviction in approximately 17% of participants, whereas the present study achieved a reduction in almost 62% of participants at post-intervention. In terms of belief flexibility, in the Ross et al. (2009) study only four out of 17 participants improved on either one (*n* = 3) or two (*n* = 1) of the three items between pre- and post-training, whereas in the present study, six out of 13 participants gave flexible responses to all three items at post-intervention. There were also promising results in terms of data gathering. Although the overall mean increase in beads to decision was greater in the Ross et al. (2009) study (mean = 2.1 beads), more detailed analysis of that study highlighted that change was greater in participants who did not JTC from the outset (mean = 4.00, sd = 4.04), with a much smaller improvement in participants who showed the JTC bias at baseline (mean = 0.44, sd = 2.13). In contrast, the present study found a slightly greater increase in beads to decision between baseline and post-intervention periods in those participants who showed the JTC bias at baseline (mean = 1.17, sd = 1.91), in comparison to those who did not JTC (mean = 0.57, sd = 1.21).

We expected that the lengthier programme with the addition of more real-life scenarios and interactive elements would help participants to generalise training points to their own experiences. This is supported by some of the comments made during completion of the training and outcome measures. Further, participants’ comments both during and at post-training suggested that the addition of real-life scenarios and the use of multimedia to illustrate key points were particularly useful and engaging. This aspect of the package was designed to illustrate how people can JTC in the real world, with the aim of helping participants to generalize key training points to their own delusional experiences, in addition to the more abstract tasks used by Ross et al. (2009). This did appear to be the case, with participants often commenting on the personal relevance of particular examples, but finding it helpful and less distressing to think about more neutral explanations. This idea of helping patients to think of a greater number of explanations for their experiences has appeared helpful in previous research. For example, Levine, Barak, and Granek (1998) suggest that their group intervention was able to target delusional conviction through first creating a state of cognitive dissonance and then helping participants to find a range of feasible explanations in addition to the delusional one.

It should be noted that the study was designed as an initial exploratory study, with the aims of establishing the acceptability and feasibility of the training programme. Although a control period was included in order to assess the stability of key outcome measures before the intervention and thus provide greater confidence that change post-intervention was not as a result of the instability of the variables being measured, the study did not include an independent control group and was not powered to detect change in key outcomes. This will clearly be crucial in future evaluation of the programme. There were a number of additional limitations to the present study, which should be addressed in future evaluation. As the study was a small pilot, there was no independent assessor: the assessments and therapy were carried out by the same researcher. Whilst they conducted the assessments according to protocol, there is a possibility that the results were influenced by experimenter bias and/or demand characteristics. Future studies would benefit from the use of a blind, independent assessor for both qualitative and quantitative outcomes. Secondly, due to the nature of the sample, whereby over 60% of participants were recruited via a local team specialising in CBTp, the majority of participants already had some experience of CBT. It is difficult to assess how this may have impacted on the results. On one hand it suggests that previous CBT did not lead to long-term reductions in particular delusional beliefs, however it may mean that the sample recruited were more socialised to the CBT model and may have benefitted more than a sample with little or no experience of CBT. Again, this possibility should be addressed in future studies, particularly as it will determine whether the programme could be used as a stand-alone therapy or as part of a course of CBT.

Not all participants showed quantitative changes in key outcomes or qualitative changes in their reasoning styles following training. This outcome has been found in previous work targeting delusional beliefs, where only a proportion of participants were found to reduce conviction following cognitive therapy (Jakes, Rhodes, & Turner, 1999). It will be interesting to explore reasons for this in future studies. For example, several participants reported qualitative changes in less strongly held delusions, which were not formally rated on measures in the present study. Therefore, it may be useful to assess and rate multiple delusions, where present, in future work in order to gain a greater understanding of any changes which may occur following training. Further, the current sample was too small to control for certain factors, such as intellectual functioning, although inspection of the data did not appear to suggest that this was a likely factor. However there has been some suggestion in the literature that IQ may play a role in JTC (e.g Van Dael et al., 2006).

Overall, the results suggest that the programme holds promise in changing, over a single session, outcomes which are typically resistant to standard treatments. Additionally, the programme is relatively easy to administer and may hold potential to be delivered by ‘non-expert’ staff following brief training. As an uncontrolled case series, the results provide a preliminary analysis which will guide future research, including providing an initial estimate of effect sizes for future larger-scale studies. Taking delusional conviction and belief flexibility as the most clinically relevant outcomes, and comparing between two independent groups (*p* = 0.05; power = 80%), a sample size of at least 26 per group would be required to reliably detect change (Cohen, 1992). If similar positive effects are found, it may be that the programme is used in combination with more generic CBT for psychosis interventions in order to improve overall efficacy.

## Figures and Tables

**Table 1:** Clinical and demographic information: means (SD) and numbers of participants (*n* = 13).

Age	44.6 (10.2) years
Sex	7 Male 6 Female
Ethnicity	6 (46.2%) White 4 (30.8%) Black African 1 (7.7%) Black Caribbean 1 (7.7%) Asian 1 (7.7%) Other
Diagnosis	7 (53.8%) Schizophrenia 4 (30.8%) Schizo-Affective Disorder 1 (7.7%) Delusional Disorder 1 (7.7%) Unspecified Nonorganic Psychosis
Duration of illness	16.73 (9.1) years
Self-reported duration of primary delusion	13.75 (7.5) years
Previous experience of CBT	12 (92.3%)
Medication	13 (100%)
Participants showing the JTC bias	6 (46.2%)
Full scale IQ	88.93 (16.4)
DASS total score	55.23 (24.02)
PANSS positive symptom scale	22.92 (3.90)
PSYRATS total score	17.77 (2.68)

Key: DASS = Depression Anxiety Stress Scales; PANSS = Positive and Negative Syndrome Scale; PSYRATS = Psychotic Symptoms Rating Scale.

**Table 2:** Mean scores (sd) and numbers of participants on key outcomes across time points.

	Baseline 1	Baseline 2	Post-intervention 1	Post-intervention 2
Probabilistic reasoning task: mean no. of beads requested (sd)	4.08 (3.17)	3.92 (2.81)	4.69 (3.01)	5.00 (3.00)
Probabilistic reasoning task: number showing JTC bias (% of total)	6 (46.2%)	5 (38.5%)	3 (23.1%)	3 (23.1%)
Mean % conviction (sd)	99.15 (2.76)	97.31 (8.32)	80.77 (31.22)	85.69 (27.99)
Belief flexibility: mean PBM (sd)	8.65 (11.29)	13.08 (27.50)	30.77 (31.48)	23.85 (22.19)
Belief flexibility: no. of participants showing positive RTHC (% of total)	1 (7.7%)	3 (23.1%)	5 (38.5%)	10 (76.9%)
Belief flexibility: number of EoE (% of total)	2 (15.4%)	3 (23.1%)	5 (38.5%)	6 (46.2%)

Key: JTC = Jumping to Conclusions; PBM = Possibility of Being Mistaken; RTHC = Reaction to Hypothetical Contradiction; EoE = Belief Flexibility.

**Table 3:** Mean Scores (sd) and statistical analysis of key outcomes across baseline and post-intervention periods (*n* = 13).

	Baseline	Post-intervention	*F*	Df	*P*	Effect size (Cohen’s d^∗^)
No. of beads requested	4.00 (2.94)	4.85 (2.75)	3.96	1, 12	0.07	0.30
% Conviction	98.23 (4.62)	83.23 (18.58)	8.52	1, 12	0.01	1.06
Belief flexibility: PBM	10.87 (16.62)	27.31 (22.04)	11.07	1, 12	0.01	0.82

Key: PBM = Possibility of Being Mistaken. ∗Cohen’s d calculated using group means & pooled SD (Dunlap et al., 1996).

**Table 4:** Examples of participant comments on the Training Programme.

Immediately after training (Post-intervention 1) *‘The programme made me question things more. I know I do JTC and will try not to in the future. I know to ask questions first and that there could be other explanations.’* *‘I learnt a lot of things – I have to gather a lot of evidence before I JTC. I can jump to negative conclusions, but there could be an innocent solution. I should look at possible positive and neutral solutions and not allow negative emotions to rule over me.’* *‘I’ve never really thought about JTC before and it highlighted how easy it is to JTC in life. I will try to take my time in judging a situation and try to get all the facts first.’* *‘It was quite simple. I learned to slow down and think carefully about the situation and be more hesitant. In the future I will be very hesitant about coming to a fixed conclusion.’* Follow-up (Post-intervention 2) *Examples of Positive Change:* *‘I noticed myself jumping to conclusions. A woman was walking round the estate. Under normal circumstances I would have found this really dodgy, but I asked a neighbour who’d been out gardening and she told me she was looking for her lost cat. I felt a lot better then.’* *‘When I see people laughing and talking about me I try thinking that I’m jumping to conclusions. When I see people with mobiles I’ve been trying to give them the benefit of the doubt – they might be taking pictures of me, but they might not. It’s less distressing thinking like this…’* *‘I have tried to think about things rationally – I’m not that important, it would be too expensive (to be under surveillance). There are issues with liberties and civil rights. The noises I hear could be a sign I’m under surveillance, but they could also be hallucinations or the neighbours.’* *‘I’ve been trying to slow down my thinking and be a bit more careful with my thoughts…before I react to something. Perhaps it is to do with my brain and what I’m thinking rather than the devil. Or it could be a day dream or something bigger that is controlling me e.g. God or aliens.’* *Examples of No Change:* *‘I’m definitely under surveillance. I’m paying double for drugs and being cheated. The police are involved and they want me to know they’re following me. I saw a security van and there’s no other reason they would be there.’* *‘I feel that people are definitely laughing at me and talking about me. I see this for myself, and they were quiet until I came by. I’m totally convinced.’*
